# Evaluation of biodistribution and safety of adenovirus vector containing MDR1 in mice

**DOI:** 10.1186/1756-9966-29-1

**Published:** 2010-01-04

**Authors:** ZhenZhen Zhao, Wei Liu, YuXi Su, Jin Zhu, GaiHuan Zheng, Qing Luo, XianQing Jin

**Affiliations:** 1 Surgery and Oncology Laboratory, Pediatric Research Institution, Children's Hospital of ChongQing Medical University, No136, 2nd Street ZhongShan, District Yuzhong, ChongQing, China; 2 Department of General Surgery, Children's Hospital of ChongQing Medical University, No 136, 2nd Street ZhongShan, District Yuzhong, ChongQing, China; 3 Department of Pathology, Children's Hospital of ChongQing Medical University, No136, 2nd Street ZhongShan, District Yuzhong, ChongQing, China; 4 Department of Hematology, Children's Hospital of ChongQing Medical University, No 136, 2nd Street ZhongShan, District Yuzhong, ChongQing, China

## Abstract

**Background:**

The aim of this study is to examine the safety and distribution of Ad-EGFP-MDR1, an adenovirus encoding human multidurg resistance gene (human MDR1), in the mice colon carcinoma model.

**Methods:**

After bone marrow cells (BMCs) were infected with Ad-EGFP-MDR1, they were administered by intra bone marrow-bone marrow transplantation (IBM-BMT). Total adenovirus antibody and serum adenovirus neutralizing factor (SNF) were determined. Biodistribution of Ad-EGFP-MDR1 was detected by in situ hybridization and immunohistochemistry. The peripheral hematocyte white blood cell (WBC), haemoglobin (Hb), red blood cell (RBC) and platelet (Plt) counts were analyzed.

**Results:**

Neither total adenovirus antibody nor SNF increased weeks after BMT. In situ hybridization and immunohistochemistry demonstrated concordant expression of human MDR1 and P-gp which were found in lung, intestine, kidney and BMCs after BMT, but not detected in liver, spleen, brain and tumor. No significant abnormality of the recovery hematocyte was observed on Day 30 after treatment.

**Conclusion:**

The results indicate that IBM-BMT administration of a replication defective adenovirus is a feasible mode of delivery, allowing exogenous transference. The findings in this study are conducted for the future long-term studies of safety assessment of Ad-EGFP-MDR1.

## Introduction

Colorectal cancer is one of the most commonly occurring malignancies in the world. It is sensitive to chemotherapy and possible to be completely remitted remission of it is possible by surgical procedure removal, the prognosis of advanced or relapsed colorectal cancer is not satisfactory[1]. Discovered some 40 years ago, Fluorouracil (FU) is still the most extensively studied drug and is considered to be the standard treatment in colorectal cancer especially in advanced cancer[2]. In recent years, 5-fluorouracil (5-Fu), leucovorin, oxaliplatin and cisplatin combination chemotherapy is one of the most effective regimen in advanced colon cancer[3]. But the dose-limiting toxicities associating with these drugs, including nephrotoxicity, myelosuppression and neurotoxicity, influence the therapeutic efficacy[4]. Some researchers found that the success of high-dose chemotherapy (HDCT) and hematopoietic stem cell transplantation in the treatment of malignancies would achieve long term complete responses because of the dose-response relationship. [5,6]

Our preliminary studies in mice indicated that, transfection of MDR1 retroviral vectors resulted in a significant increase in P-gp expression in murine bone marrow cells, transplantation of which into recipients resulted in consistently high levels of MDRl expression in developing hematopoietic cells after treatment and protecting the normal BMCs from the toxic effects of anticancer drugs[7,8].

In this study, we evaluated the tissue distribution and safety of IBM-BMT applied Ad-EGFP-MDR1 in short term. The BMCs were infected with the adenovirus vector with multiplicity of infection (MOI) 50 in 30 μl, and transplantated in tumor-bearing Balb/c mice. Serum total adenovirus antibody, serum adenovirus neutralizing factor (SNF), hematological, histopathology and distribution of human MDR1 were determined to make sure the extent of response caused by this treatment.

## Materials and methods

### Mice

Balb/c mice (female and male are half-and-half, 16 g-18 g of weight) were purchased from animal center of ChongQing Medical University and maintained under specific pathogen-free conditions until use in animal facilities. Their housing, care, diet and maintenance conformed to the guidelines of China, the "regulation for the care and use of laboratory animals".

### Cell lines

The murine colon carcinoma cell line CT26 and human embryonic kidney (HEK) 293 cell lines (SIBCB, China) were cultured at 37°C and 5% CO2 in RPMI 1640 (Gibco, America) medium, containing 10% fetal calf serum (PAA, Austria).

### Preparation of donor BMC and IBM injection of BMCs

The BMCs were collected from the femurs and tibias of Balb/c mice and injected via IBM, as described in[9], and then cultured at a density of 2 × 10^5^ cells/ml in IMDM media supplemented with bovine serum albumin and L-glutamine. Cultures were stimulated with a combination of the cytokines, thrombopoietin (10 ng/ml), flt3-ligand (10 ng/ml), stem cell factor (20 ng/ml), granulocyte colony-stimulating factor (15 ng/ml), interleukin (IL)-3 (10 ng/ml) and IL-6 (25 ng/ml). (R&D, America). Cells were populated two days after primary culture, and co-cultured with Ad-EGFP-MDR1 (kindly presented by professor Tong-Chuan He, EGFP:enhanced green fluorescence protein) (MOI 50) for two days. The cultured cells were washed by phosphate buffered sodium (PBS) for three times and harvested. Total RNA of cells was isolated and qualitatively analyzed with a reverse transcription polymerase chain reaction (RT-PCR) kit (Takara, Japan). For MDR1: forward primer: AAAGCTGTCAAGGAAGCCAA, reverse primer: ACTCCATCATCGAAACCAGC. For beta-actin, forward primer: AAGTGTGACGTTGACATCCG, Reverse primer: GAAAGGGTGTAAAACGCAGC. Reverse transcriptase reaction was performed with PCR conditions were as follows: 94°C for 2 min (1 cycle); followed 94°C for 20 sec, 55°C for 1 min, 72°C for 30 sec (30 cycle); followed 72°C for 10 min(1 cycle). P-gp activity was determined by using the daunomycin efflux assay and detected by fluorescence microscope. P-gp in BMCs was investigated by western bolt. BMCs were washed once with PBS and lysed in lysis buffer. The protein concentration was determined by BCA protein assay (Pierce). The cellular fractions were subjected to SDS-PAGE [10% (w/v)] gel. The separated proteins were electroblotted on polyvinyliden difluoride (PVDF) membranes (Millipore), which were then washed once with Tris buffered saline containing Tween 20 (TBS-T), and then blocked in blocking buffer for 2 h. After washing with TBS-T, the membranes were probed with antibodies (Santa Cruz) at a dilution of 1:1000 in TBS-T. After three washes with TBS-T, membranes were treated for 1 h with HRP-conjugated, indicated antibodies diluted to 1:10,000 in TBS-T. After three washes with TBS-T, immunoreactive protein bands were revealed with an ECL Western blot analysis system (Bio-Rad). Films were scanned and analyzed with Quantity One software (Bio-Rad). In addition cell viability was assessed with a trypan blue dye exclusion test. Cell quantification was carried out using a haemocytometer and an optical microscope. The successful infected BMCs with green fluorescence were determined by flow cytometry.

The donor BMCs were injected from the femurs into the bone marrow cavity using a microsyringe containing the donor BMCs (2 × 10^6^/30 μl). Anesthesia for transplantation: the mice were given Sumianxin (a mixture of xylidinothiazoline, edathamil, dihydroetorphine hydrochloride and haloperidol) (AMMS, China) 0.5 ml/kg via intramuscular injection. At the end of the transplantation the mice were observed from the anesthesia.

### Experimental protocols

Mice were randomly assigned to four groups, 20 animals in each. For establishment of tumors, Balb/c mice were injected with 5 × 10^7^/ml, 100 μl CT 26 cells into the right armpit. 10 days after injection, the tumor size was detected by ultrasound, then chemotherapy was started with 25 mg/kg 5-FU via intraperitoneal injection once a day for 5 days, a week constituting one therapeutic course and with 0.02 mg/kg vincristine via intraperitoneal at the first day of each week.

Mice in Group A were tumor-bearing and transplanted with the transfected MDR1-BMCs via IBM-BMT (Tumor+chemotherapy+MDR1-IBM-BMT). Mice in Group B were tumor-bearing and transplanted untreated BMCs via IBM-BMT (Tumor + chemotherapy + IBM-BMT). Mice in Group C were no tumor with the MDR1-BMCs via IBM-BMT and chemotherapy (No tumor + chemotherapy + MDR1-IBM-BMT). Group D was prepared as control, in this group PBS was used instead of tumor xenograft, transplantation and chemotherapy (No tumor + No tranplatation + No chemotherapy). On the second day after the end of 5-Fu chemotherapy in the first week, the mice were transplanted with BMCs by IBM injections.

### Posttransplantation management

75% Alcohol and gentamycin were administered to the surgical wound everyday for one week. Each mouse was observed once every morning throughout the transplantation for changes in general appearance and behavior. Body weights were measured twice a week. Food consumption was qualitatively assessed daily for each group.

Blood samples were collected via caudal vein once pretreatment and on Day 1, 3, 14 and 30 posttransplantation for analysis by automated hematology analyzer (KX-21, Sysmex Inc, Japan).

### Detection of human MDR1 gene biodistribution

Mice were necropsied on Day 3, 7, 14, 21 and 30, with three samples necropsied at one time. And the following tissues were collected: bone marrow, brain, heart, liver, kidneys, spleen, lungs and intestine. Tumors were also collected from the group A and B. Tissues were taken macroscopic examination and preserved in neutral-buffered 10% formalin. After 48 hours, the tissues were embedded in paraffin, stained with hematoxylin and eosin, and microscopically examined. A tissue microarray (TMA) was constructed (6 mm ×4 μm). Two duplicate specimens from each sample were placed on the array. Paraffin-embedded sections were stained with standard immunohistochemical techniques as introduced in [10].

In situ hybridization experiments were carried out with a mixture of specific digoxin-labeled oligonucleotide anti-sense probe for the human MDR1 (TBD, China). The MDR1 DNA probe consisted of the fragment corresponding to nucleotides 514-482 of the human MDR1 mRNA (genebank accession number AF016535). ISH signals were scored with a fluorescence microscope (Olympus BX51, Tokyo, Japan). In situ hybridization was performed on paraffin-embeds tissue sections according to the manufacturer's protocol. The positive signal for human MDR1 was detected with fluorescein isothiocyanate. Consecutive tissue sections were also hybridized with sense probe under the same conditions.

### Detection of Adenovirus-specific antibody and Serum neutralizing factors (SNF)

Adenovirus-specific antibody levels were evaluated by ELISA on Day 3, 7, 14 days after transplantation. Diluted serum samples were added to 96-well microtitier plates coated with the protein of adenovirus. Each sample had duplicate determination, tetramethylbenzidin were added to produce a colored reaction. The absorbance was read at 450 nm with a reference filter of 650 nm with the microplate reader.

To detect SNF against Ad-EGFP-MDR1, serum was incubated at 55°C for 30 min to inactivate complement. 2 × 10^5^/well HEK 293 cells were plated into 24-well plates (BD, America) and incubated for four hours before sample dilution. Serum was incubated with equal volume of Ad-EGFP-MDR1 (MOI 10) for 1 hour at 37°C. The serum/Ad-EGFP-MDR1 mixtures were transferred onto the HEK293 cell and incubated 4 hours, supernatant was removed and fresh medium was added. The green fluorescence of cells was measured with flow cytometry at 24 hours after incubation. [11]

### Statistical analysis

Hematology and ELISA results were expressed as mean ± standard error (S.E). Data were analyzed using unpaired student's *t*-test, or one-way analysis of variance ANOVA with SAS (Biostatistics department, Chongqing Medical University). Significance was set at *P *< 0.05.

## Results

### The transfected BMCs and macroscopic observations

**Figure 1:**
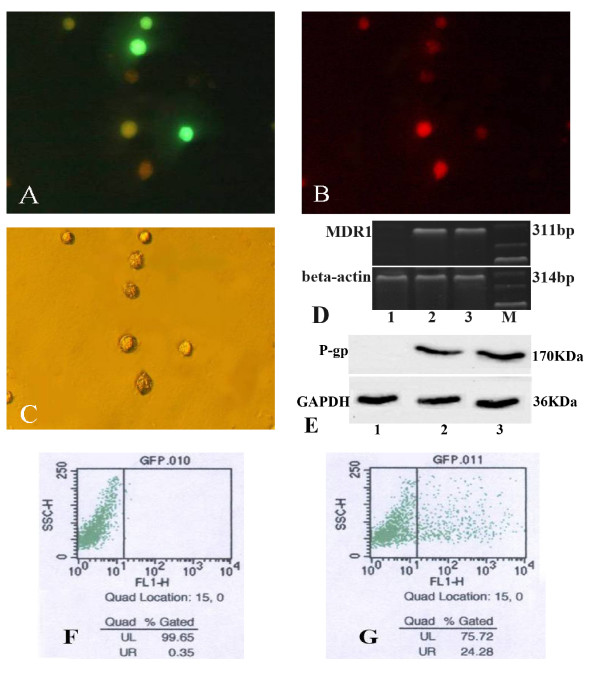
**BMCs infected with Ad-EGFP-mdr1**. Daunomycin efflux assay detected by fluorescence microscope. A: BMCs incubated with Ad-EGFP-MDR1 for 48 h expressed EGFP(green). × 400 B: Daunomycin (red) aggregated inside the BMCs which were not infected with the adenovirus. × 400 C: Bright field images of those BMCs × 400. MDR1 mRNA in BMCs was detected by RT-PCR. D: The expected size band of human MDR1 mRNA was 311 bp. The expected size band of mouse beta-actin was 314 bp. The expression of P-gp in BMCs was assessed by western blot. E: Ad-EGFP-mdr1 infection induces expression and production of human P-gp in BMCs. Flow cytometry determined percentage of green fluorescence. BMCs infected with Ad-EGFP-mdr1 successfully would show green under fluorescence channel analyses. F: the background was about 0.4%. G: The infection rate of BMCs incubated with Ad-EGFP-mdr1 for 48 h was about 24.3%, 1.BMCs. 2.BMCs incubated with Ad-EGFP-mdr1 for 48 h. 3. Positive control. M:marker.

Expression of EGFP was detected by fluorescence microscope to observe the infection efficiency of Ad-EGFP-MDR1 on BMCs. The subsequent distribution of the daunomycin was also monitored by the fluorescence microscope. Most of daunomycin aggregated inside the BMCs which were not infected with the adenovirus. MDR1 could effectively express in cells infected with Ad-EGFP-MDR1 and reduce the accumulation of daunomycin. (Figure 1) MDR1 mRNA highly expressed in the treated BMCs which showed a unique MDR1 specific band compared with the untreated cells. (Figure 1) We studied the effects of Ad-EGFP-MDR1 on BMCs. An increase in the BMCs expression of P-gp was seen. (Figure 1) Every group of BMCs cultured was low viability losses, maintaining cell culture viability above 88% [see Additional file 1]. BMCs infected with Ad-EGFP-mdr1 successfully would show green under fluorescence channel analyses by flow cytometry. The infection rate of BMCs incubated with Ad-EGFP-MDR1 was obviously higher than that of control group. The infection rate of BMCs incubated with Ad-EGFP-mdr1 for 48 h was about 24.3%, and the background was about 0.4%.

About 10-12 days after injection, a neoplasm size from 3 mm × 3 mm × 4 mm to 5 mm × 5 mm × 7 mm appeared in the axillary area of mice in group A and B [see Additional file 2]. Then the mice became inactive and had reduced food consumption 1 month after transplantation. And the relative tumor weights in group A and B significantly increased. Two mice died in group B and one in group A, and the remaining mice of these two groups survived for more than 2 months.

The appearance of lung, liver and spleen changed in group A and B at the advanced stage. The thoracic cavity and venous drainage were compressed by the grown neoplasm, which led to splenomegaly, enlargement of the liver and hydrothorax.

### Histopathology

Morphology examination was performed on Day 3, 7, 14, 21, 30 after transplantation. Mice showed no significant pathological changes in the brain, lung, heart, kidneys, spleen, liver and intestine at the first week after posttreatment. However, on Day 21 and 30, lesions including cardiac dilatation, congested lungs and hydrothorax occurred in mice in group A and B. At the same time, mild hydropic degeneration was found in the centrilobular regions of liver lobules, mild lymphoid and megakaryocytic hyperplasia was shown in the spleen, ascites and abnormalities of central nervous system and digestive system were not manifested. Histology was normal for mice in group C and D.

### Immunobiology

**Figure 2:**
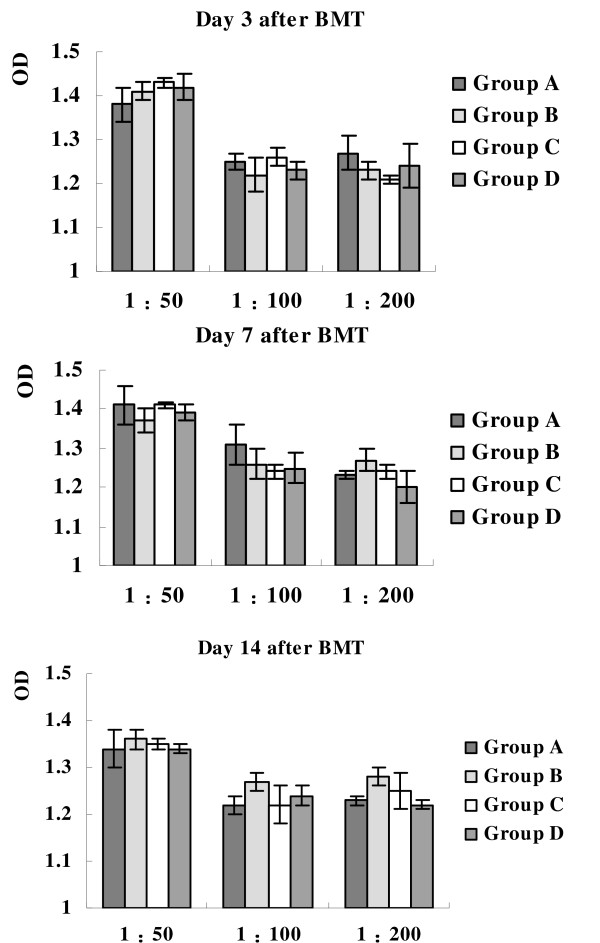
**Adenovirus-specific antibody measured by ELISA**. Optical density (OD) of group A and C had no significant difference with that of group B and D. It could be inferred that the levels of adenovirus-specific antibody of group A and C did not increase on Day 3, 7, 14 after transplantation. The error bars represent one standard deviation from the mean values. These results are representative of three independent experiments.

The levels of adenovirus-specific antibody were measured by ELISA. Optical density (OD) of group A and C had no significant difference with that of group B and D. (Figure 2) [see Additional file 3] It could be inferred that the levels of adenovirus-specific antibody of group A and C did not increase on Day 3, 7, 14 after transplantation.

**Figure 3:**
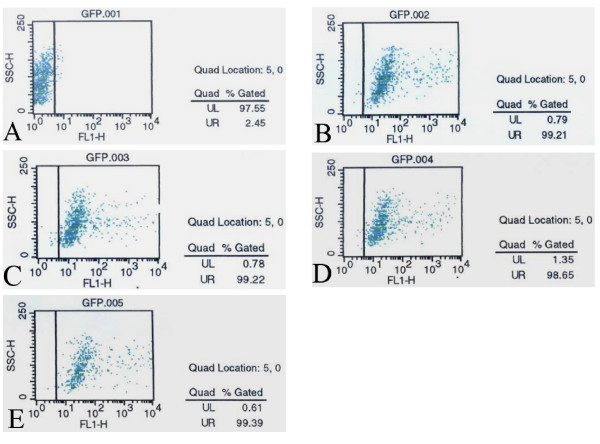
**SNF was detected by measuring the fluorescent intensity of HEK293 cells using a flow cytomtry**. A: The background was 2.45%. The percentages of green fluorescent cells were 99.21%(B), 99.22%(C), 98.65%(D) and 99.39%(E) for group A to D respectively on Day 7 after the treatment. Fluorescence intensity of infected HEK 293 cells was inversely proportional to SNF level. SNF against Ad-EGFP-MDR1 was not detected in all groups.

Fluorescence intensity of infected HEK 293 cells, which was measured with a flow cytometry, was inversely proportional to SNF level. The SNF could inhibit the infection efficiency of Ad-EGFP-MDR1 and result in the reduction of the fluorescence intensity. However, almost all samples were infected, the percentages of green fluorescence (infected BMCs) were 99.21%, 99.22%, 98.65% and 99.39% for group A to D respectively on Day 7 posttransplantation. The background was 2.45%. (Figure 3) [see Additional file 4] We inferred that SNF against Ad-EGFP-MDR1 was not detected in all groups.

### Tissue distribution of Ad-EGFP-MDR1

**Figure 4:**
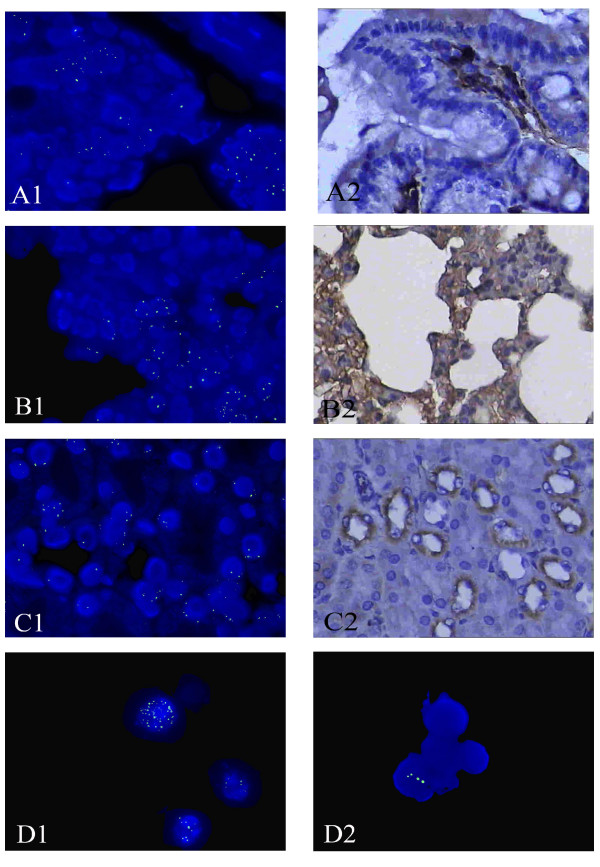
**Tissue distribution of Ad-EGFP-MDR1 in group A**. The expression of P-gp (brown staining) in group A on Day 14 after BMT by immunohistochemistry. (A2, B2, C2)×400. In situ hybridization localized Human MDR1 expression in the tissues of group A on Day 14 after BMT. (A1, B1, C1, D) MDR1 DNA was labeled with FITC (green signals). ×1000. P-gp and MDR1 DNA predominantly expressed in intestine (A), lung (B), kidney (C) and the BMCs (D1), but they were not detected in the liver, spleen, brain and tumor tissues. Human MDR1 still could be detected in the BMCs in group A on Day 30 posttreatmen(D2).

**Figure 5:**
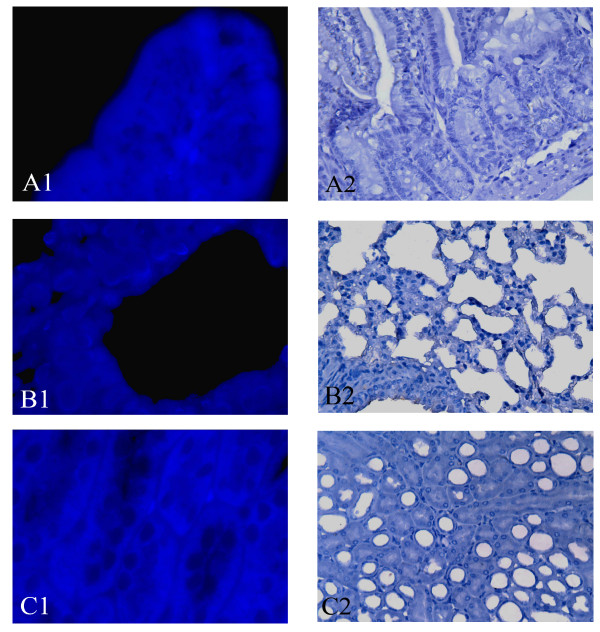
**Tissue distribution of Ad-EGFP-MDR1 in group B**. The expression of P-gp (A2, B2, C2 ×400) and MDR1 DNA (A1, B1, C1×1000)in group B on Day 14 after BMT were not detected in intestine, lung and kidney.

Tissue distribution of Ad-EGFP-MDR1 was assessed by immunohistochemistry and in situ hybridization. Transgene expression was detected at higher frequency in necroscopy of the major tissue of all groups. On Day 7 after BMT, expression of human MDR1 and P-gp could be detected in kidney, lung and intestine of mice in group A and C (Figure 4). [see Additional file 5] And it was higher obviously on day 14 and lower on day 30 (Figure 4), while not detected in any tissue of group B and D at any time (Figure 5). Human MDR1 predominantly expressed in bone marrow cells and could still be detected on Day 30 posttreatment, but its expression was not detected in the liver, spleen, brain and tumor tissues in our study.

### Hematology analysis

There were some changes in hematology parameters. In group A and C, white blood cell (WBC) counts, haemoglobin (Hb), red blood cell (RBC) counts and platelet (Plt) counts decreased after 3 days of IBM-BMT. But only WBC counts in group C at that time had statistically significant difference compared with group D (*P *<0.05). WBC counts and Plt counts in group A increased as the tumor's growthing. It could be inferred that the tumor might stimulate myelopoiesis and cause a leukemoid reaction. However, at the end of first chemotherapy they decreased with statistical significance (*P *< 0.05). On Day 30 after BMT, the counts of peripheral hematocyte in group A and C were close to that in group D, and no significant morphological abnormality was found in the recovering hematocyte. [see Additional file 6] It demonstrated that the transplantation of MDR1-BMCs was able to reconstitute the hematopoietic system.

## Discussion

It was demonstrated that the efficacy of human MDR1 for chemoprotection permitted the intensified chemotherapy in experimental animals[12]. Retroviral vector was used in our previous study, but in this research the recombinant adenovirus vector was used for the reason that retroviral vector may cause carcinogenesis[13]. It was reported that platinum chemotherapeutic agents are used to treat many types of cancer, but drug resistance to platinum chemotherapy is multifactorial[14]. So vincristine, which was used in chemotherapy of gastroenteric tumor and a substrate of P-gp, was used in this study.

While a variety of models have been used to evaluate the safety of adenovirus-mediated gene therapy[15,16], and some of them have been clinical application[17], previous studies had demonstrated that administration of adenovirus was associated with dose-limiting toxicity, pathology and immunogenicity. In this study, we administered the adenovirus vector by infecting BMCs via IBM-BMT. By in situ hybridization and immunohistochemistry analysis, human MDR1 and P-gp were found in lung, intestine and kidney of both genders of colon carcinoma mice in group A and C. The biodistribution of human MDR1 by in situ hybridization was consistent with the expression of P-gp.

In previous study, the concentration of adenovirus receptor in the liver was high, so as the distribution of adenovirus vector[18]. Some studies on the homing behavior of hemopoietic stem cells showed that part of the transplanted cells stayed in spleen for a time, [19] while others reported the number of donor cells in spleen kept at a low level at all times in nonablated mice[20]. In our study, human MDR1 and P-gp were not detected in liver and spleen of any group. Maybe there were not enough niches in our study. In further research human MDR1 would be detected by taking shorter time, such as 12 hours or 1 day after transplantation and be analyzed through more sensitive methods.

Some study reported that systemically administered adenovirus vector had been shown inhibition of myeloid progenitor growth, inducing transient leucopenia and thrombocytopenia[21]. In this study, our data of blood cell counts did not support a role for MDR1-BMCs in dysregulated haemopoiesis in short term posttranplantation.

It had been reported that adenovirus vectors eliciting the humoral immune response for many years[22]. And many factors would influence immune responses, like route of administration, dose of vector, host and so on. In this study, Day 7 after BMT was chosen to investigate the humoral response after administration, because some researchers reported that SNF increased and reached peak levels at Day 7 after local administration[11]. Our results showed that no SNF was detected after transplantation and the levels of adenovirus-specific antibody also had no significance among each group. It indicated that the adenovirus vector did not have notable effect on immune response. In some other studies, adenovirus vector were administered by intravenous injection, intra-arterial injection or localized delivery routes[23]. Adenovirus was detected in the injection site or major organs[24], proinflammatory cytokine was also detected in the serum, and inflammatory response appeared at and near the site of injection. Their data showed that SNF and anti-adenovirus antibody levels had been elevated postadministration[25,26]. We considered that these differences were caused by the differences of delivery routes. Studies with IBM-BMT showed it induced persistent donor specific tolerance in mice even if the radiation doses were reduced to sublethal levels. And it was good for allogeneic BMT, because no GVHD developed, no graft failure occurred when the radiation dose was low, and hemopoietic recovery was rapid[27].

## Conclusions

Enhanced BMCs clearance of pharmaceuticals via P-gp may reduce plasma concentrations and in turn the therapeutic efficacy of these agents. It remained technically feasible that drug resistance gene was able to protect haemopoiesis from the side effects of cytotoxin in chemotherapy[28]. Safety studies have arisen, and the possibility of an adverse long-term effect of Ad-EGFP-MDR1 transfer on BMCs would be an important content in the further study. In conclusion, our result shows there has no serious side effect of adenovirus MDR1 gene therapy in short term, which provide useful baseline data for future long-term studies aimed at evaluating the safety of Ad-EGFP-MDR1.

## Competing interests

The authors declare that they have no competing interests.

## Authors' contributions

XQJ designed the experiments. ZZZ drafted the manuscript. ZZZ, WL and YXS performed the experiment. JZ, GHZ and QL carried out the statistical analysis and data interpretation.All authors read and approved the final manuscript.

## Supplementary Material

Additional file 1 **Trypan blue dye exclusion test**. BMCs inviable were dyed by trypan blue. Every group of BMCs cultured was low viability losses, maintaining cell culture viability above 88%. A: BMCs with Ad-EGFP-MDR1. B: BMCs with PBS Click here for file

Additional file 2 **Colon carcinoma detected by ultrasound**. (A) The xenograft tumor in armpit was detected by ultrasound after 10 days of CT26 tumor cell injection. It was about 3 mm × 5 mm × 5 mm. (B) The blood vessel of the neoplasm. The speed of arterial blood was 0.017 m/s. Click here for file

Additional file 3 **Summary of immunobiology evaluations of adenovirus-specific antibody levels by ELISA**. OD of group A and C had no significant difference with that of group B and D. Adenovirus-specific antibody did not increased at 3, 7, 14 days after transplatation in group A and C. Click here for file

Additional file 4 **SNF detected reversely with green fluorescent of HEK293**. SNF on Day 3,7,14 after transplantation was detected by measuring the fluorescent intensity of HEK293 cells using a flow cytometry. SNF against Ad-EGFP-MDR1 was not detected in all groups. Click here for file

Additional file 5 **Tissue distribution of Ad-EGFP-MDR1**. The expression of P-gp by immunohistochemistry in group A on Day 14 after BMT. A to H, ×400. Samples were counterstained with hematoxylin, the brown staining indicating P-gp. In situ hybridization localized Human MDR1 expression in the tissues of group A Day 14 after BMT. A1 to H1, ×1000. MDR1 DNA was labeled with FITC (green signals). P-gp and MDR1 DNA expression could be detected in intestine (B), lung (C) and kidney (D), also in the BMCs (I), but they were not expressed in tumor (A), heart (E), liver (F), spleen (G) and brain (H). Human MDR1 still could be detected in BMCs of group A on Day 30 posttreatment. Click here for file

Additional file 6 **Peripheral blood cell analyzed by hematology analyzer**. In group A, C and D, WBC (A), RBC (B), Plt (C) and (Hb) (D) were decreased after 3 days of IBM-BMT. But only WBC in group C at that time had statistical significance compared with group D (*P *< 0.05). WBC and Plt in group A were increased after the tumor growth and at the end of first chemotherapy they were decreased with statistical significance (*P *< 0.05). And on Day 30 after BMT, the counts of peripheral hematocyte in group A and C were close to that in group D. Click here for file
