# Supplementary material for: Evaluation of biodistribution and safety of adenovirus vector containing MDR1 in mice
Source: J Exp Clin Cancer Res. 2010 Jan 4;29(1):1. doi: 10.1186/1756-9966-29-1 (PMC2819043; doi:10.1186/1756-9966-29-1)
Supplement: Additional file 1 — Trypan blue dye exclusion test. BMCs inviable were dyed by trypan blue. Every group of BMCs cultured was low viability losses, maintaining cell culture viability above 88%. A: BMCs with Ad-EGFP-MDR1. B: BMCs with PBS [file 1756-9966-29-1-S1.DOC]

| **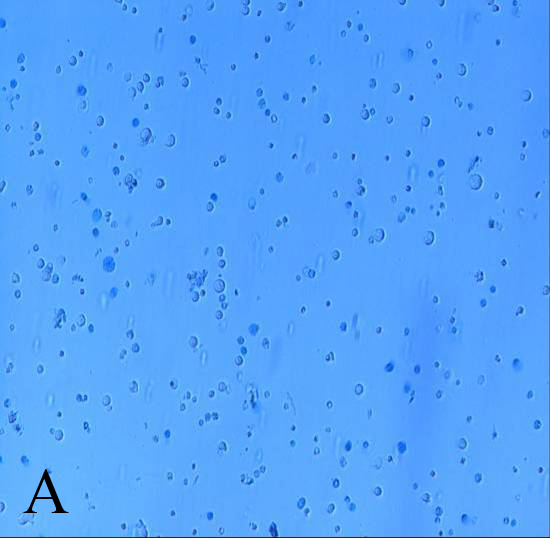** | **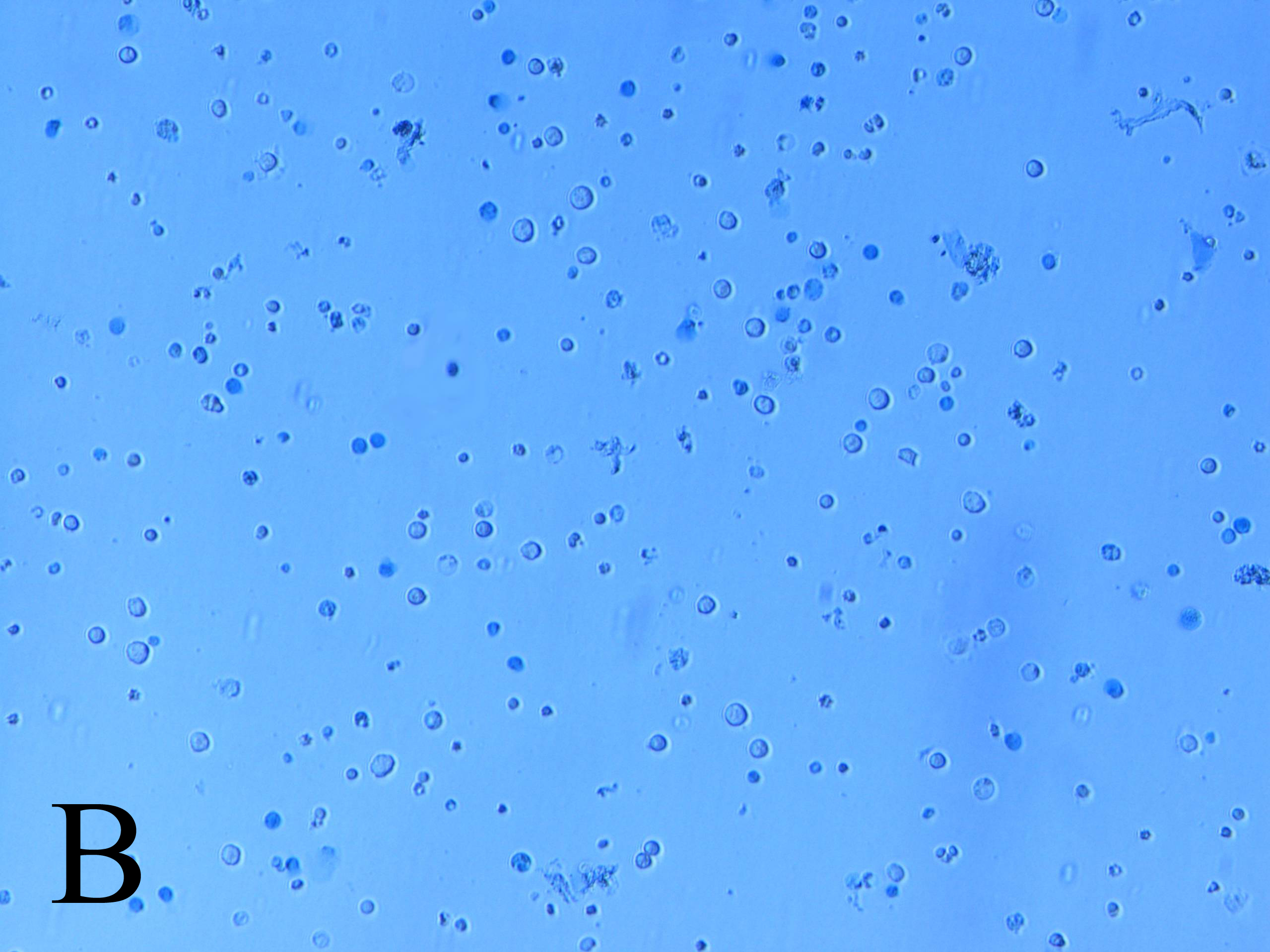** |
| --- | --- |

Table BMCs viability by Trypan Blue dye exclusion test. （%, $\overline{x}$±s, n=3）

| groups | BMCs with Ad-EGFP-MDR1 | BMCs with PBS | Control group |
| --- | --- | --- | --- |
| BMCs viability | 88.22±2.64 | 90.40±2.36 | 90.79±2.67 |

**1: Trypan blue dye exclusion test.** BMCs inviable were dyed by trypan blue. Every group of BMCs cultured was low viability losses, maintaining cell culture viability above 88%. A: BMCs with Ad-EGFP-MDR1. B: BMCs with PBS
