# Supplementary material for: Evaluation of biodistribution and safety of adenovirus vector containing MDR1 in mice
Source: J Exp Clin Cancer Res. 2010 Jan 4;29(1):1. doi: 10.1186/1756-9966-29-1 (PMC2819043; doi:10.1186/1756-9966-29-1)
Supplement: Additional file 2 — Colon carcinoma detected by ultrasound. (A) The xenograft tumor in armpit was detected by ultrasound after 10 days of CT26 tumor cell injection. It was about 3 mm × 5 mm × 5 mm. (B) The blood vessel of the neoplasm. The speed of arterial blood was 0.017 m/s. [file 1756-9966-29-1-S2.doc]

| **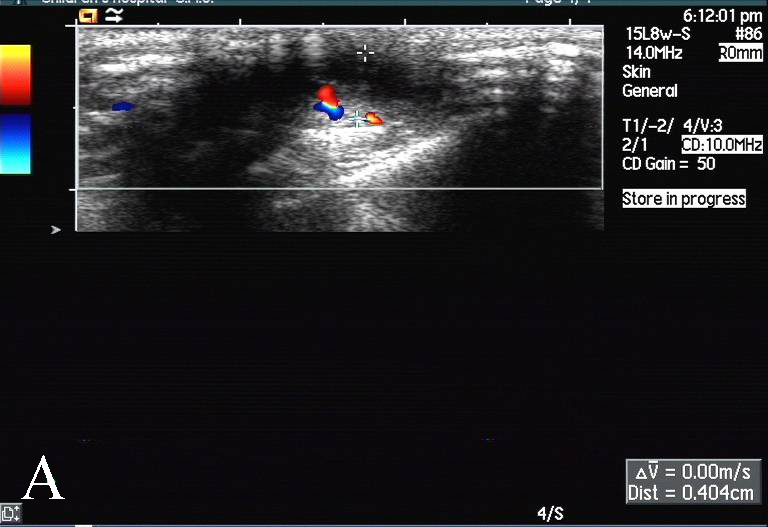** | **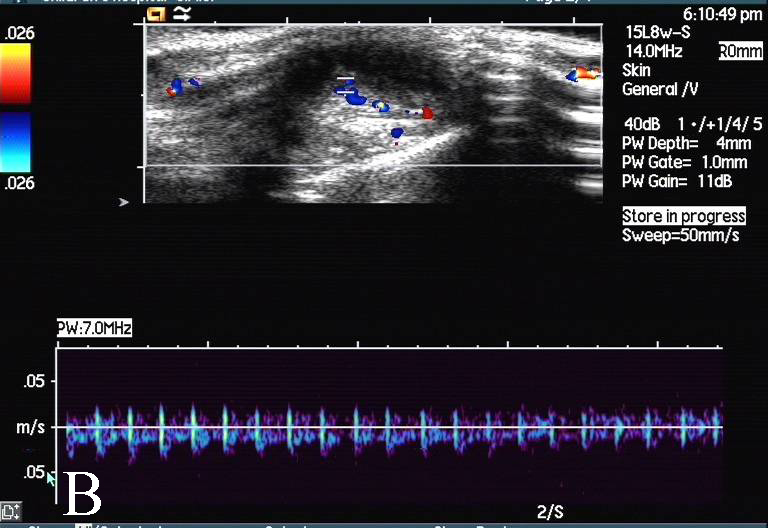** |
| --- | --- |

**2: Colon carcinoma detected by ultrasound.** (A)The xenograft tumor in armpit was detected by ultrasound after 10 days of CT26 tumor cell injection. It was about 3 mm ×5 mm ×5mm. (B) The blood vessel of the neoplasm. The speed of arterial blood was 0.017m/s.
