# Supplementary material for: Evaluation of biodistribution and safety of adenovirus vector containing MDR1 in mice
Source: J Exp Clin Cancer Res. 2010 Jan 4;29(1):1. doi: 10.1186/1756-9966-29-1 (PMC2819043; doi:10.1186/1756-9966-29-1)
Supplement: Additional file 3 — Summary of immunobiology evaluations of adenovirus-specific antibody levels by ELISA. OD of group A and C had no significant difference with that of group B and D. Adenovirus-specific antibody did not increased at 3, 7, 14 days after transplatation in group A and C. [file 1756-9966-29-1-S3.doc]

|  | group | 1:50 | 1:100 | 1:200 |
| --- | --- | --- | --- | --- |
| D3after BMT | A | 1.35±0.04 | 1.25±0.02 | 1.07±0.04 |
|  | B | 1.41±0.02 | 1.22±0.04 | 1.03±0.02 |
|  | C | 1.43±0.01 | 1.26±0.02 | 1.01±0.01 |
|  | D | 1.47±0.03 | 1.23±0.02 | 1.04±0.06 |
| D7after BMT | A | 1.41±0.05 | 1.31±0.05 | 1.10±0.01 |
|  | B | 1.37±0.03 | 1.26±0.04 | 1.07±0.03 |
|  | C | 1.41±0.01 | 1.24±0.02 | 1.04±0.02 |
|  | D | 1.39±0.02 | 1.25±0.04 | 1.00±0.04 |
| D14after BMT | A | 1.34±0.06 | 1.22±0.02 | 1.10±0.01 |
|  | B | 1.38±0.02 | 1.27±0.02 | 1.08±0.02 |
|  | C | 1.35±0.01 | 1.22±0.04 | 1.05±0.04 |
|  | D | 1.31±0.01 | 1.24±0.02 | 0.97±0.01 |

**3: Summary of immunobiology evaluations of adenovirus-specific antibody levels by ELISA.** OD of group A and C had no significant difference with that of group B and D. Adenovirus-specific antibody did not increased at 3, 7, 14 days after transplatation in group A and C.
