# Supplementary material for: Evaluation of biodistribution and safety of adenovirus vector containing MDR1 in mice
Source: J Exp Clin Cancer Res. 2010 Jan 4;29(1):1. doi: 10.1186/1756-9966-29-1 (PMC2819043; doi:10.1186/1756-9966-29-1)
Supplement: Additional file 4 — SNF detected reversely with green fluorescent of HEK293. SNF on Day 3,7,14 after transplantation was detected by measuring the fluorescent intensity of HEK293 cells using a flow cytometry. SNF against Ad-EGFP-MDR1 was not detected in all groups. [file 1756-9966-29-1-S4.doc]

|  |
| --- |
| SNF detected reversely with green fluorescent of HEK293 |

**4: SNF on Day 3,7,14 after transplantation was detected by measuring the fluorescent intensity of HEK293 cells using a flow cytomtry.** SNF against Ad-EGFP-MDR1 was not detected in all groups.
