# Supplementary material for: Evaluation of biodistribution and safety of adenovirus vector containing MDR1 in mice
Source: J Exp Clin Cancer Res. 2010 Jan 4;29(1):1. doi: 10.1186/1756-9966-29-1 (PMC2819043; doi:10.1186/1756-9966-29-1)
Supplement: Additional file 5 — Tissue distribution of Ad-EGFP-MDR1. The expression of P-gp by immunohistochemistry in group A on Day 14 after BMT. A to H, ×400. Samples were counterstained with hematoxylin, the brown staining indicating P-gp. In situ hybridization localized Human MDR1 expression in the tissues of group A Day 14 after BMT. A1 to H1, ×1000. MDR1 DNA was labeled with FITC (green signals). P-gp and MDR1 DNA expression could be detected in intestine (B), lung (C) and kidney (D), also in the BMCs (I), but they were not expressed in tumor (A), heart (E), liver (F), spleen (G) and brain (H). Human MDR1 still could be detected in BMCs of group A on Day 30 posttreatment. [file 1756-9966-29-1-S5.doc]

| 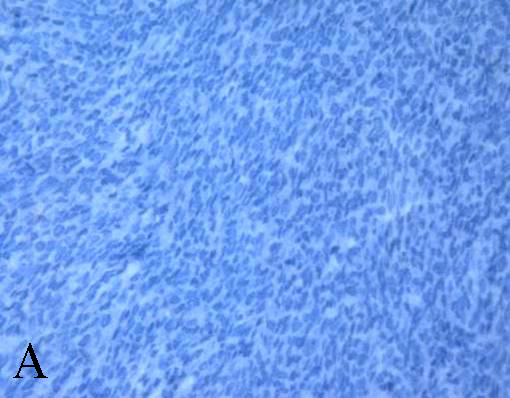 | 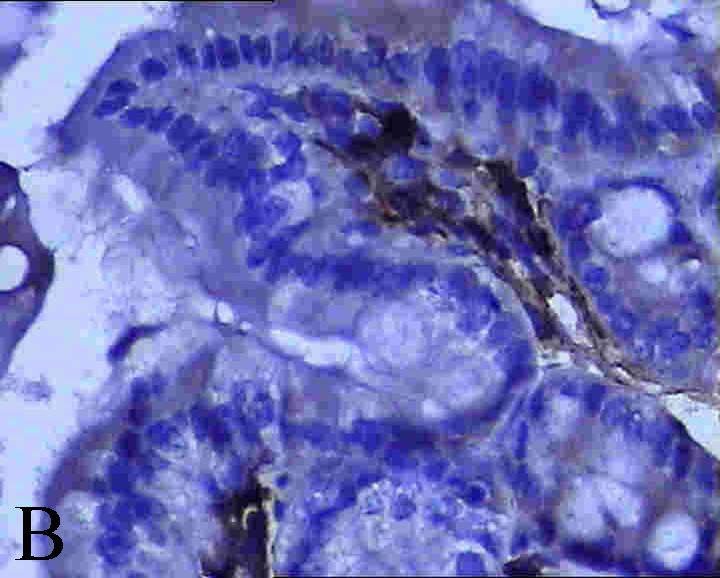 | 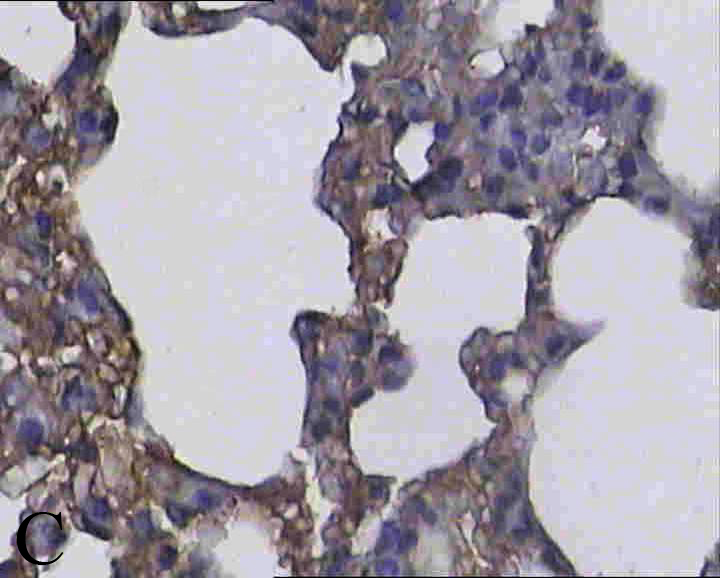 | 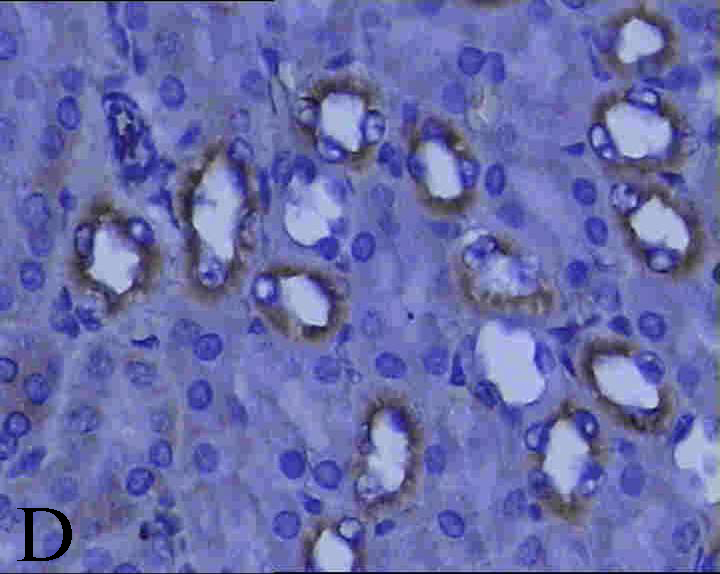 |
| --- | --- | --- | --- |
| 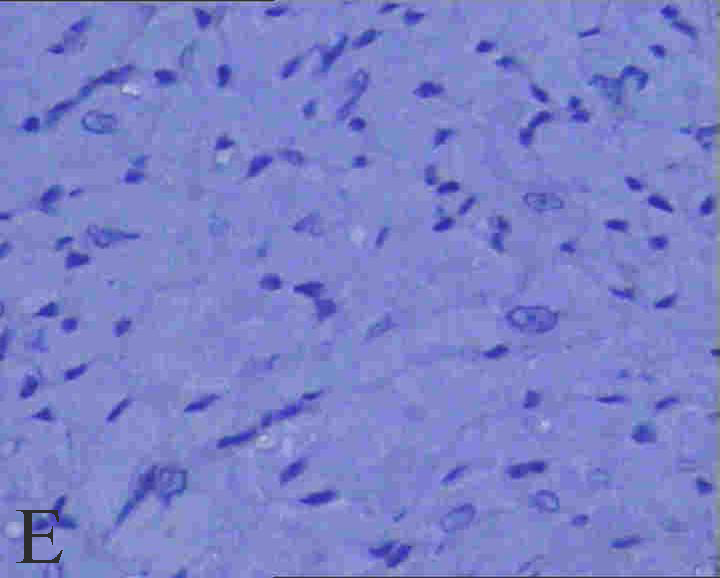 | 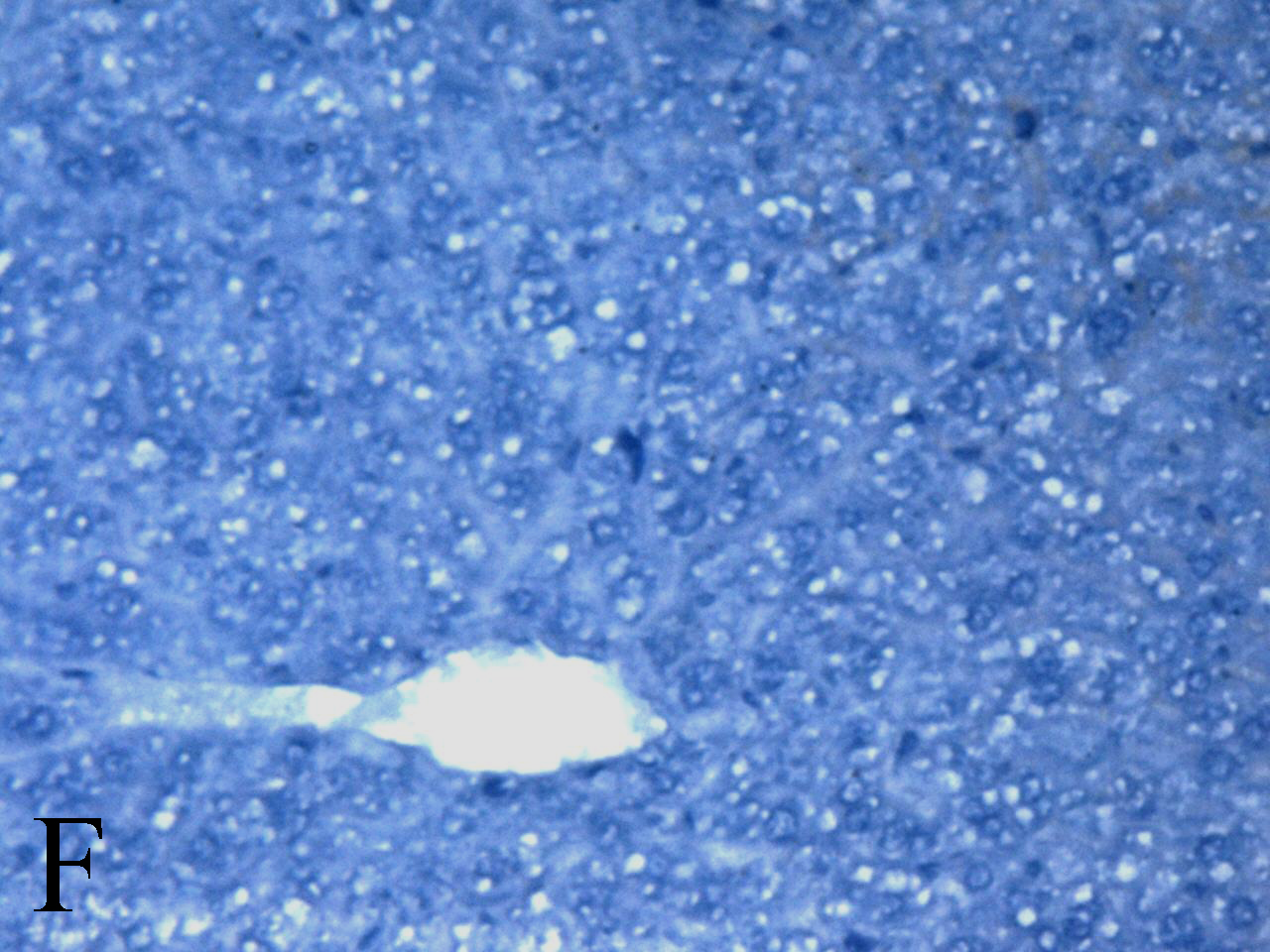 | 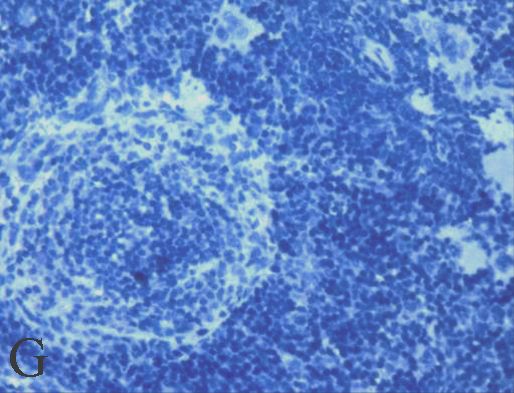 | 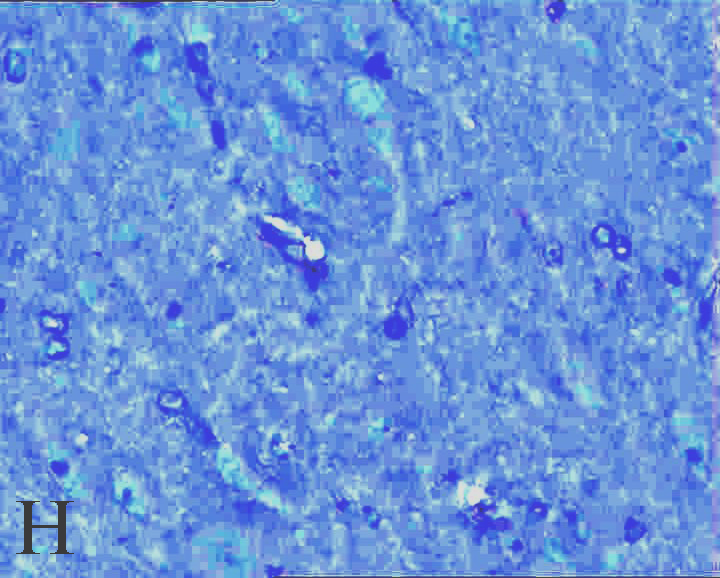 |
| **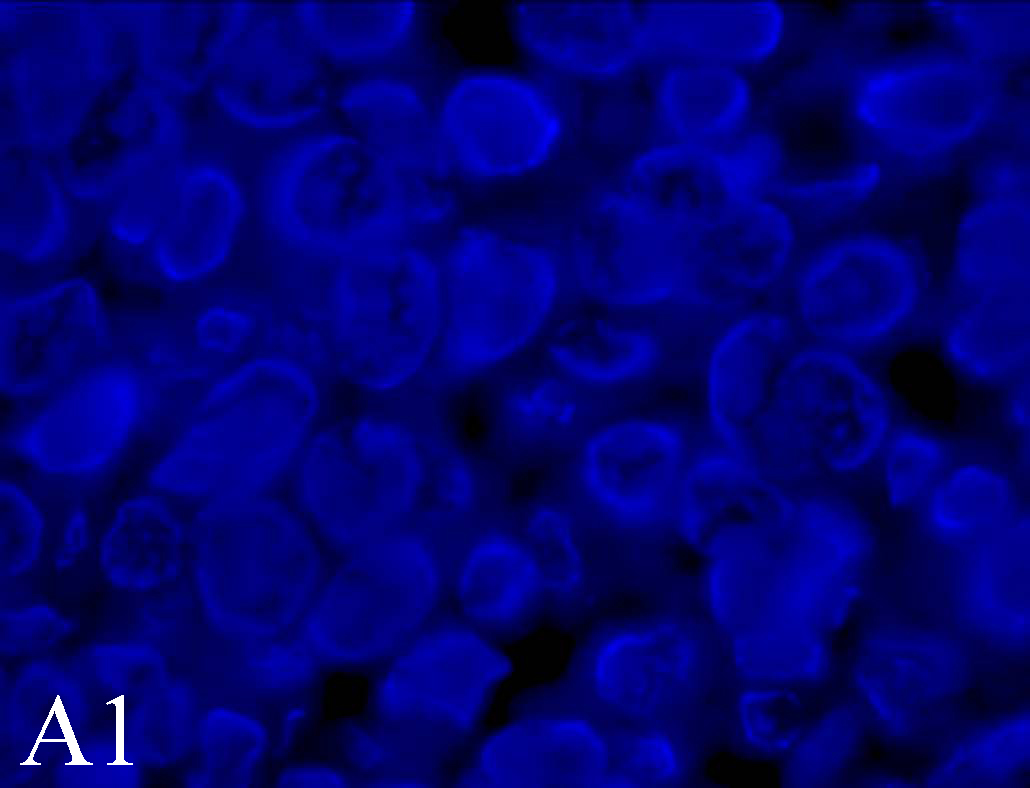** | **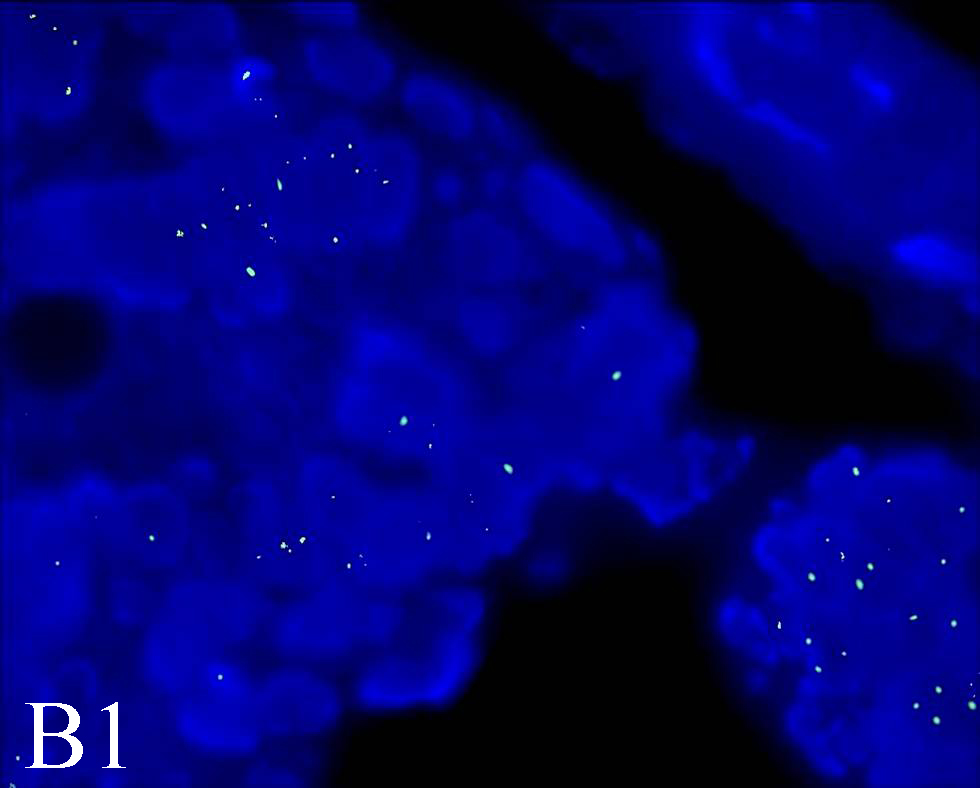** | **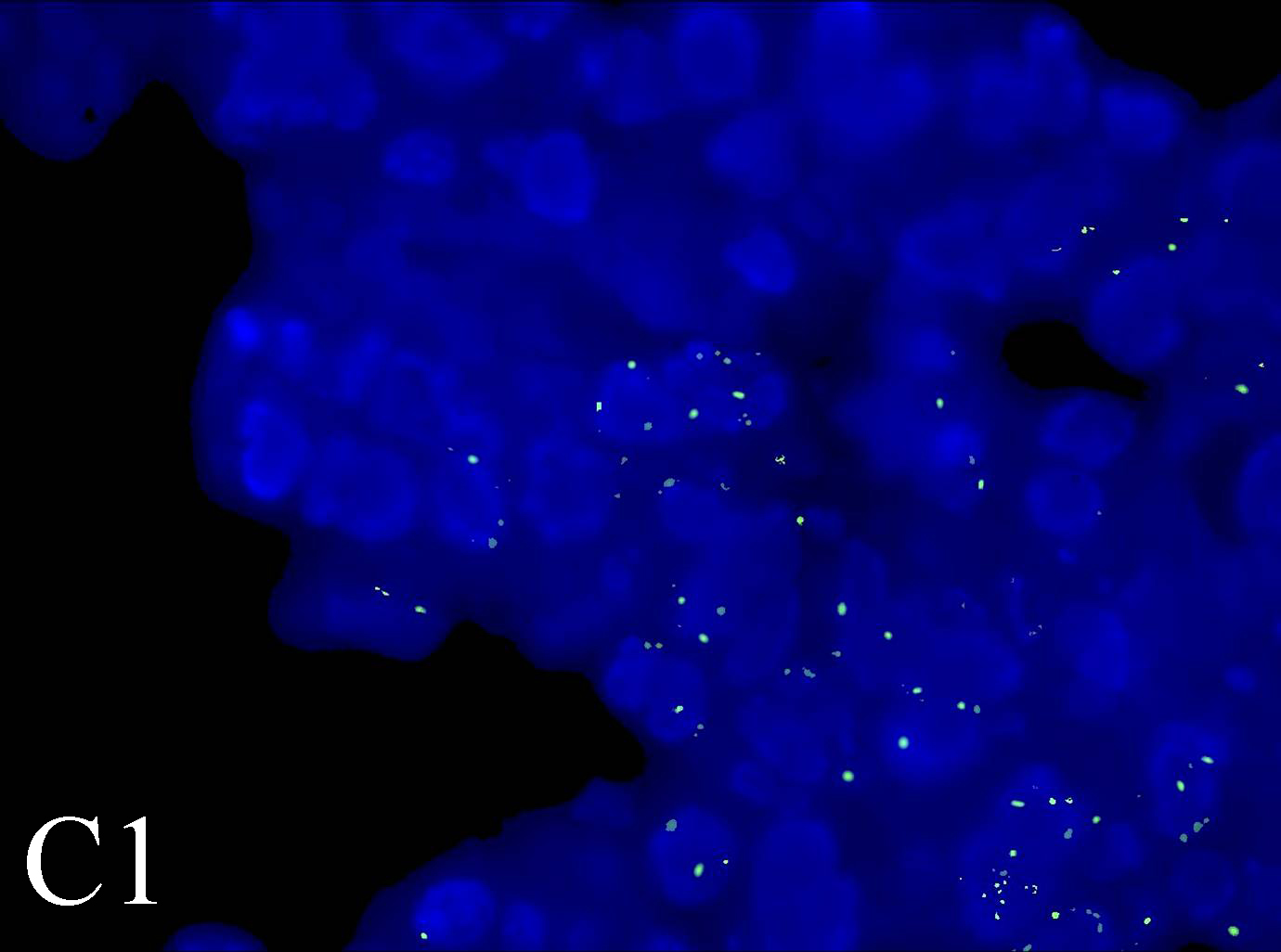** | **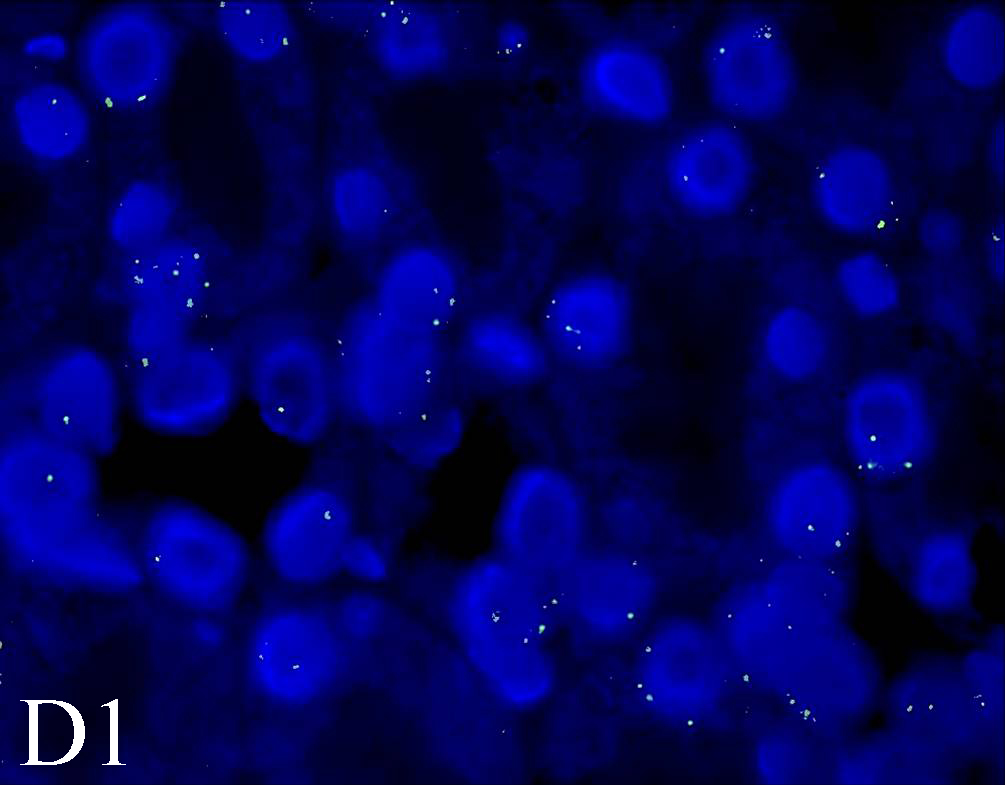** |
| **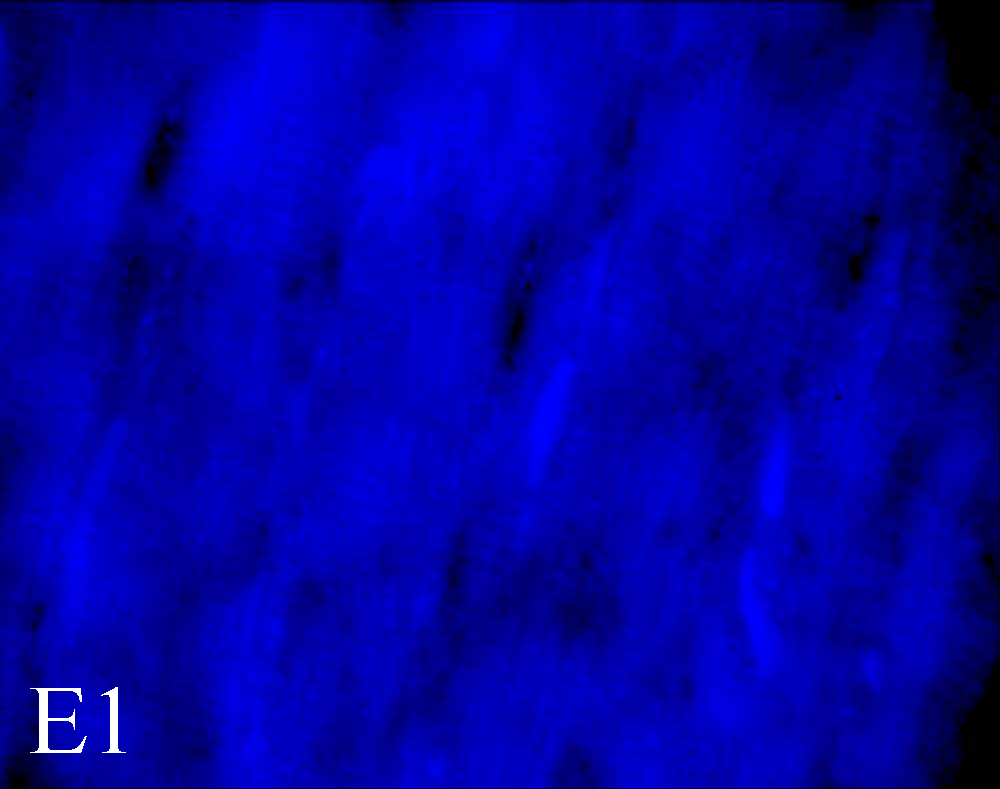** | **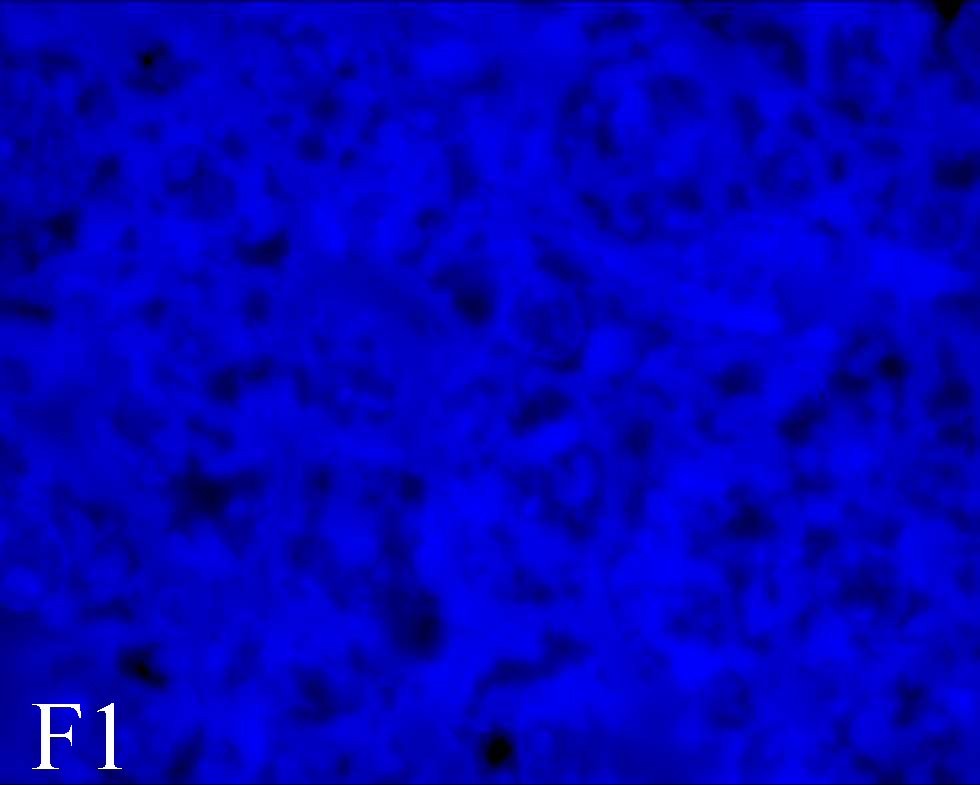** | **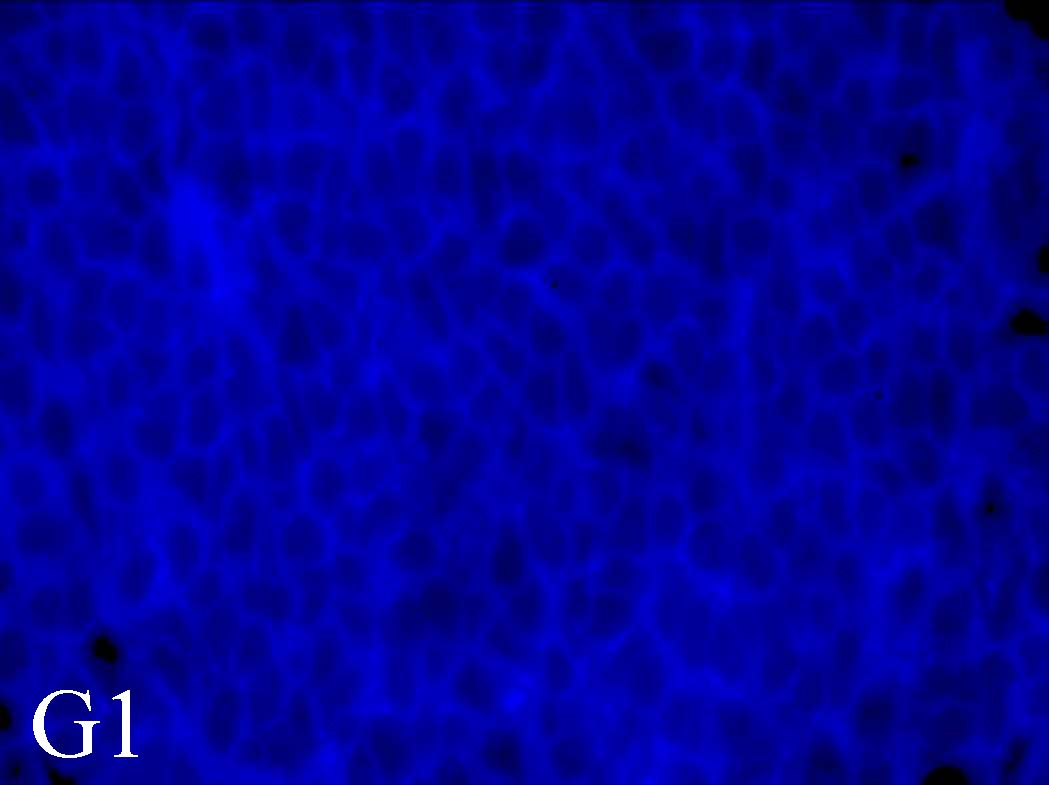** | **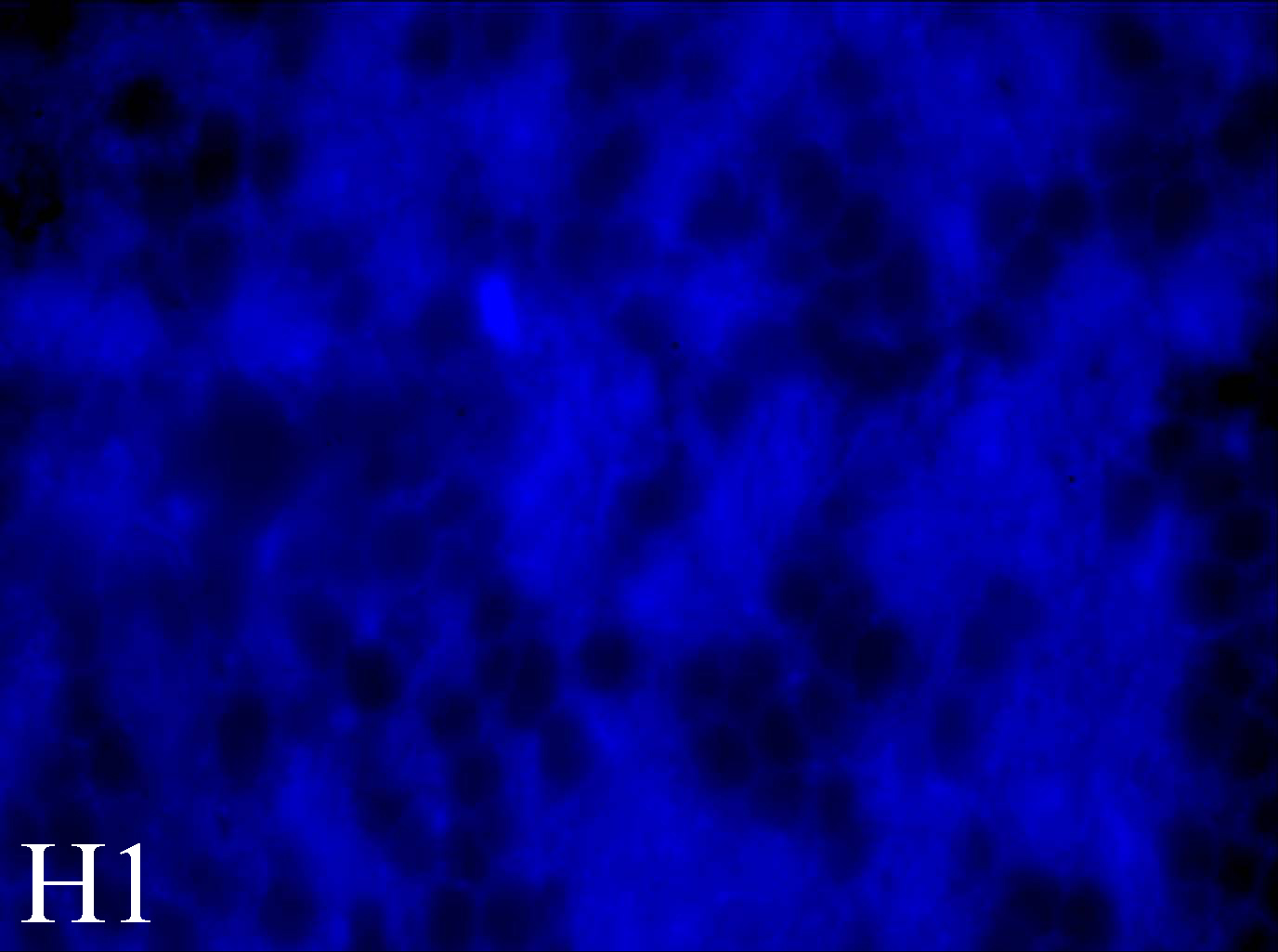** |

**5: Tissue distribution of Ad-EGFP-MDR1.**

The expression of P-gp by immunohistochemistry in group A on Day 14 after BMT. A to H, ×400. Samples were counterstained with hematoxylin, the brown staining indicating P-gp. In situ hybridization localized Human MDR1 expression in the tissues of group A Day 14 after BMT. A1 to H1, ×1000. MDR1 DNA was labeled with FITC (green signals).

P-gp and MDR1 DNA expression could be detected in intestine (B), lung (C) and kidney (D), also in the BMCs (I), but they were not expressed in tumor (A), heart (E), liver (F), spleen (G) and brain (H). Human MDR1 still could be detected in BMCs of group A on Day 30 posttreatment.
