# Supplementary material for: Evaluation of biodistribution and safety of adenovirus vector containing MDR1 in mice
Source: J Exp Clin Cancer Res. 2010 Jan 4;29(1):1. doi: 10.1186/1756-9966-29-1 (PMC2819043; doi:10.1186/1756-9966-29-1)
Supplement: Additional file 6 — Peripheral blood cell analyzed by hematology analyzer. In group A, C and D, WBC (A), RBC (B), Plt (C) and (Hb) (D) were decreased after 3 days of IBM-BMT. But only WBC in group C at that time had statistical significance compared with group D (P < 0.05). WBC and Plt in group A were increased after the tumor growth and at the end of first chemotherapy they were decreased with statistical significance (P < 0.05). And on Day 30 after BMT, the counts of peripheral hematocyte in group A and C were close to that in group D. [file 1756-9966-29-1-S6.doc]

**
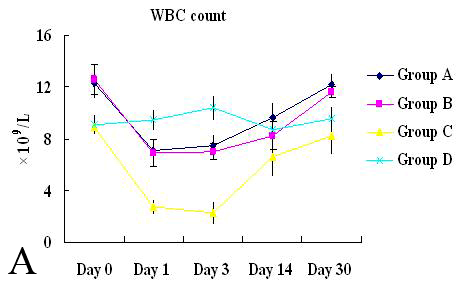

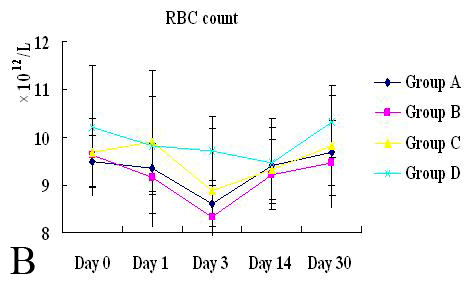
**

**
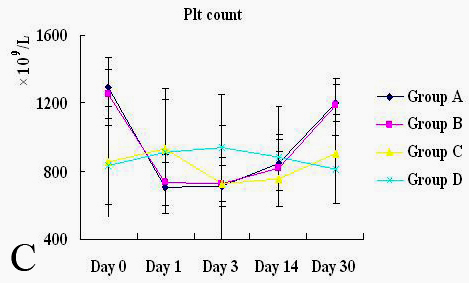

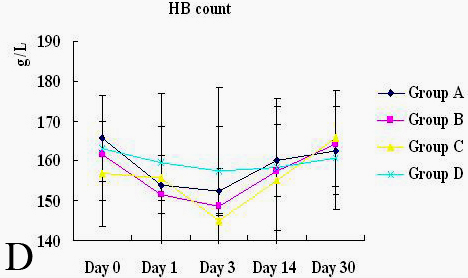
**

**6: Peripheral blood cell analyzed by hematology analyzer.** In group A, C and D, WBC (A), RBC (B), Plt (C) and (Hb) (D) were decreased after 3 days of IBM-BMT. But only WBC in group C at that time had statistical significance compared with group D (*P*<0.05). WBC and Plt in group A were increased after the tumor growth and at the end of first chemotherapy they were decreased with statistical significance (*P*<0.05). And on Day 30 after BMT, the counts of peripheral hematocyte in group A and C were close to that in group D.
